# Clinical observation of submandibular gland transfer for the prevention of xerostomia after radiotherapy for nasopharyngeal carcinoma: a prospective randomized controlled study of 32 cases

**DOI:** 10.1186/1748-717X-9-62

**Published:** 2014-02-21

**Authors:** Xiangmin Zhang, Folin Liu, Xiaolin Lan, Lijiang Yu, Wei Wu, Xiuhong Wu, Fufu Xiao, Shaojin Li

**Affiliations:** 1 Department of Head and Neck, Tumor Hospital of Ganzhou, Ganzhou, Jiangxi Province, People’s Republic of China; 2 First Affiliated Hospital of Gannan Medical University, Gannan, Jiangxi Province, People’s Republic of China; 3 Department of Stomatology, Peking Union Medical College Hospital, Chinese Academy of Medical Sciences, Beijing, People’s Republic of China; 4 Department of Radiation Oncology, Tumor Hospital of Ganzhou, Ganzhou, Jiangxi Province, People’s Republic of China; 5 Ganzhou Institute of Cancer Research, No. 19, HuaYuan Qian Road, Ganzhou 341000, Jiangxi Province, People’s Republic of China

**Keywords:** Nasopharyngeal carcinoma, Radiotherapy, Xerostomia, Submandibular gland, Transfer

## Abstract

**Background:**

The aim of this study was to evaluate the clinical efficacy of submandibular gland transfer for the prevention of xerostomia after radiotherapy for nasopharyngeal carcinoma.

**Methods:**

Using the randomized controlled clinical research method, 65 patients with nasopharyngeal carcinoma were randomly divided into an experimental group consisting of 32 patients and a control group consisting of 33 patients. The submandibular glands were averted to the submental region in 32 patients with nasopharyngeal carcinoma before they received conventional radiotherapy; a lead block was used to shield the submental region during therapy. Prior to radiotherapy, the function of the submandibular glands was assessed using imaging. Submandibular gland function was measured using 99mTc radionuclide scanning at 60 months after radiotherapy. The data in the questionnaire regarding the degree of xerostomia were investigated and saliva secretion was measured at 3, 6, 12, and 60 months after radiotherapy. In addition, the 5-year survival rate was calculated.

**Results:**

After follow-up for 3, 6, and 12 months, the incidence of moderate to severe xerostomia was significantly lower in the experimental group than in the control group. The average amount of saliva produced by the experimental and control groups was 1.60 g and 0.68 g, respectively (*P* < 0.001). After follow-up for 60 months, the uptake and secretion functions of the submandibular glands in the experimental group were found to be significantly higher than in the control group (*P* < 0.001 and *P* < 0.001, respectively). The incidence of moderate or severe xerostomia was significantly lower than in the control group (15.4% and 76.9%, respectively; *P* < 0.001). The 5-year survival rates of the experimental group and the control group were 81.3% and 78.8%, respectively, and there was no significant difference between the two groups (*P* = 0.806).

**Conclusions:**

After a 5 year follow-up period involving 32 patients who had their submandibular glands transferred for the prevention of xerostomia after radiotherapy for nasopharyngeal carcinoma, we found that clinical efficacy was good. This approach could improve the quality of life of nasopharyngeal carcinoma patients after radiotherapy and would not affect long-term treatment efficacy.

## Background

Nasopharyngeal carcinoma (NPC) is a common malignancy in the southeast provinces of China. Currently, concurrent radiochemotherapy is a mainstay of curative treatment for NPC. Xerostomia is a frequent and relevant side effect for NPC patients after radiation therapy (RT). Xerostomia, although not life-threatening on its own, affects patients’ quality of life because it causes serious disorders in tasting, chewing, and swallowing, as well as sleeping disorders. After RT, xerostomia can be treated with fluoride, pilocarpine, and some other drugs, but their efficacy is unsatisfactory [1]. Prevention of radiation-induced xerostomia can be achieved by changing the dose-time-splits to improve the biological effects of treatments, such as appropriate RT, intensity-modulated RT and RT plus protective agent [2,3]; however, most patients will not benefit because of the extra expense entailed in doing this.

In an effort to prevent radiation-induced xerostomia for patients with head and neck squamous cell carcinoma, Jha and Seiklay [4-6] first reported on the transfer of the submandibular gland to the submental space, which is an area that can be shielded from radiation without detrimental effects on cure rates in tumors distant from the submental triangle; this approach achieved good results. In a prospective randomized phase III clinical trial, a new way of preventing radiation-induced xerostomia was demonstrated, namely submandibular gland transfer; this was significantly more effective than oral pilocarpine [6]. Based on findings from animal experiments, we transferred the submandibular glands of 32 NPC patients before conventional RT to prevent xerostomia. After a follow-up period of 5 years, it was clear that we had achieved good preventative effects.

## Methods

### Ethics statement

The Ethical Committee of the Tumor Hospital of Ganzhou Review Board approved the study protocol (20040311), and the study was conducted in accordance with the principles of the Declaration of Helsinki regarding research involving human subjects. Each of the patients provided written informed consent to participate after the nature of the study had been explained to them.

### Inclusion and exclusion criteria

#### Inclusion criteria

The inclusion criteria were as follows: (1) Patients aged 20–60 years. (2) Patients who had undergone a nasopharyngeal biopsy for the diagnosis of undifferentiated nasopharyngeal non-keratinizing carcinoma. (3) Patients with no cervical lymph node metastasis or unilateral cervical lymph node metastasis. (4) Patients who had undergone conventional RT. (5) A Karnofsky performance score ≥ 80. (6) Life expectancy of at least 1 year. (7) Patients who understood and voluntarily signed an informed consent form.

#### Exclusion criteria

The exclusion criteria were as follows: (1) Bilateral cervical lymph node metastasis. (2) Bilateral district cervical lymph node metastasis. (3) Submandibular gland diseases. (4) A history of neck surgery or RT. (5) Distant metastasis. (6) Patients in poor general condition who could not tolerate surgery.

### Clinical data

This was a prospective clinical randomized controlled study. A total of 65 patients with NPC, from March 2004 to August 2006, were randomly divided into two groups; the experimental group consisted of 32 patients and the control group consisted of 33 patients. There were no significant differences between the two groups concerning the general information (Table 1). Before RT, the 32 patients in the experimental group underwent unilateral submandibular gland transfer. For those patients with unilateral cervical lymph node metastasis, the contralateral submandibular gland was transferred. For patients with no lymph node metastasis, the contralateral submandibular gland transfer was carried out according to tumor location in the nasopharynx.

**Table 1:** General clinical data for the experimental and control groups

**Parameters**	**Experimental group (n = 32)**	**Control group (n = 33)**
Age	26–60	23–60
Median age	45.6	47.8
Males	25	24
Females	7	9
Male:female ratio	3.57:1	2.67:1
Stage		
I	5	5
II	13	12
III	12	13
IV	2	3
Radiotherapy alone	18	17
Concurrent chemoradiotherapy	14	16

### Preoperative examination

In addition to a special inspection of the nasopharyngeal carcinoma, the head and neck were examined to check the wetness of the oral mucosa, the saliva pool of the mouth floor, and bilateral submandibular duct saliva secretion in the mouth. Patients in whom an impairment of submandibular gland function was expected were excluded from the study.

### Surgical methods

**Figure 1:**
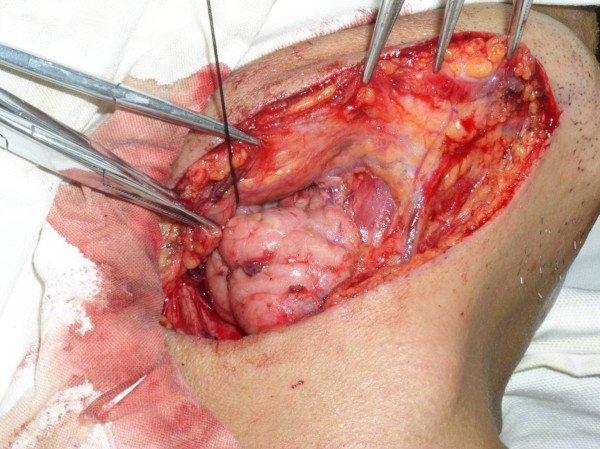
Photograph showing a 2 cm parallel incision that has exposed the submandibular gland.

**Figure 2:**
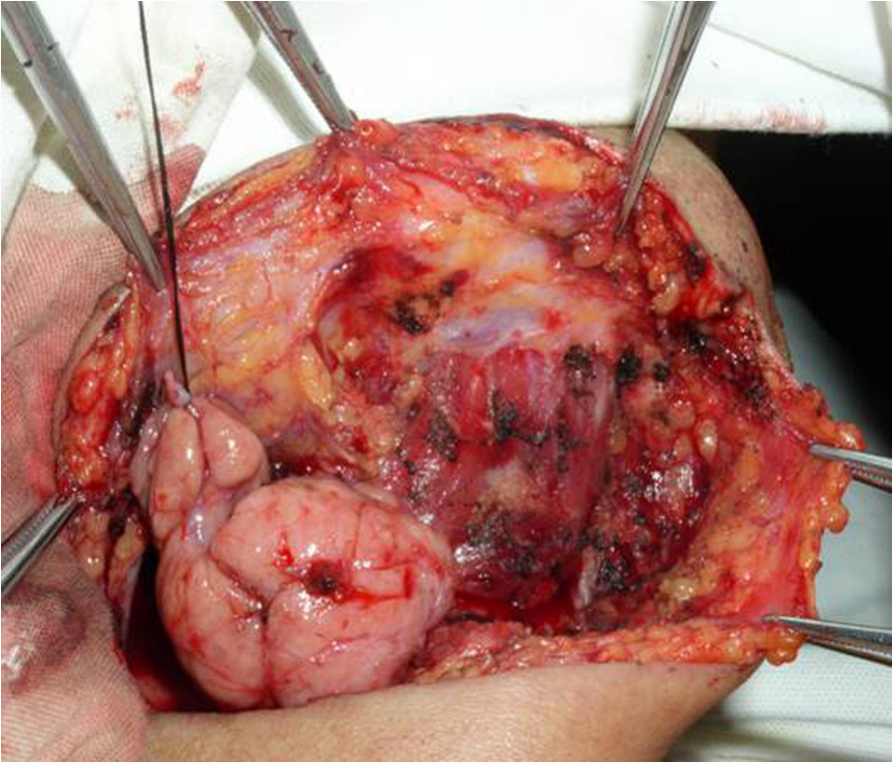
Photograph showing dissected adipose tissue.

**Figure 3:**
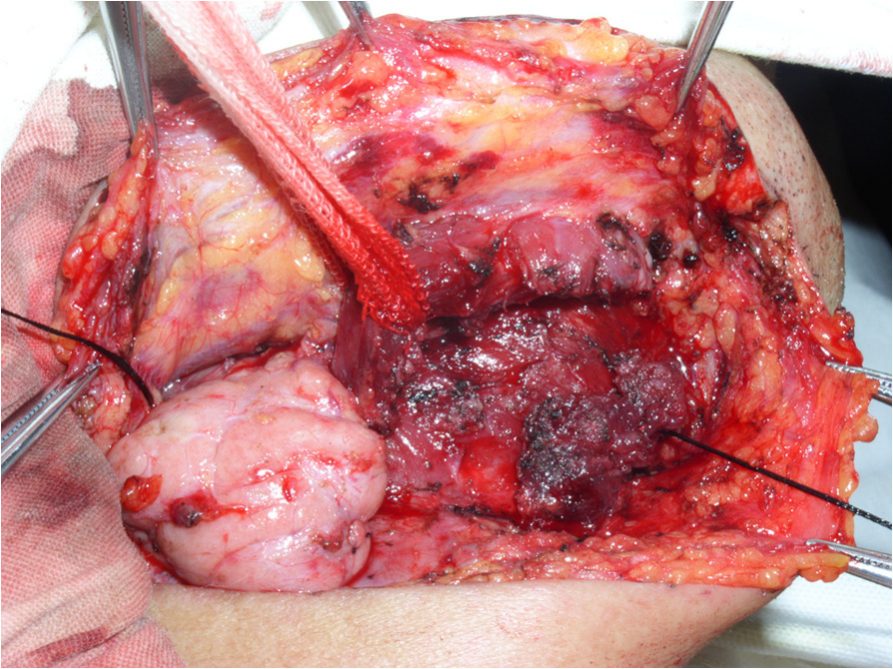
Photograph showing frozen submandibular gland and the anterior belly of the digastric.

**Figure 4:**
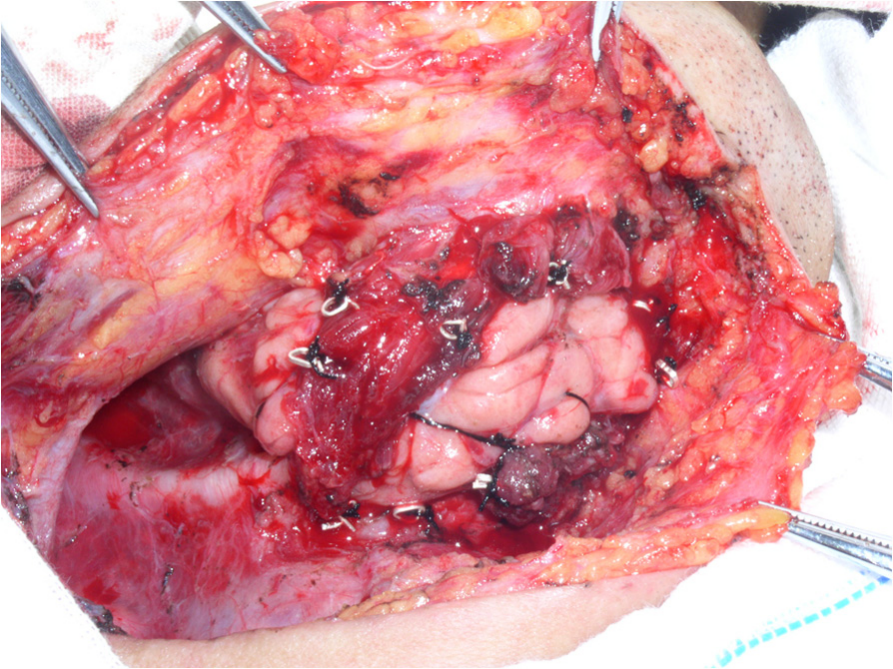
Photograph showing the submandibular gland fixed in position with silver clips that were also used as markers.

Surgery was carried out under local or general anesthesia. The following procedures were performed: (1) A 2 cm long submandibular parallel incision, a skin incision, and subcutaneous separation of a platysma flap (up and down) extending to the horizontal branch of the mandible and hyoid were undertaken; the submandibular gland was exposed (Figure 1). (2) Specimens obtained from the exploration of Zone I and Zone I lymph node adipose tissue dissection were quickly frozen and sectioned; evaluation confirmed that no cancer cell metastasis was present (Figure 2). (3) Release of the lower edge of the submandibular gland was accomplished by the following procedure: separation of the digastric belly; location of the facial artery and vein on the edge of the deep surface and their cutoff after double legation; freeing of the posterior pole of the submandibular gland while retaining the facial artery, vein, submandibular ganglion, and the marginal mandible branch of the facial nerve; loosening the edge of the submandibular gland; and freeing the anterior belly of the digastric cutoff region of the mylohyoid muscle (Figure 3). (4) The external maxillary artery, vein, and submandibular duct functioned as a pedicle, and the submandibular gland was released and transferred to the submental area of the anterior belly of the digastric deep surface; this was fixed with silver clips around the gland that acted as RT markers (Figure 4). For the use of these clinical materials for research purposes, prior patient's consent and approval from the Ganzhou Institute of Cancer Research Ethics Committee (20040311) were obtained.

### Radiation treatment

**Figure 5:**
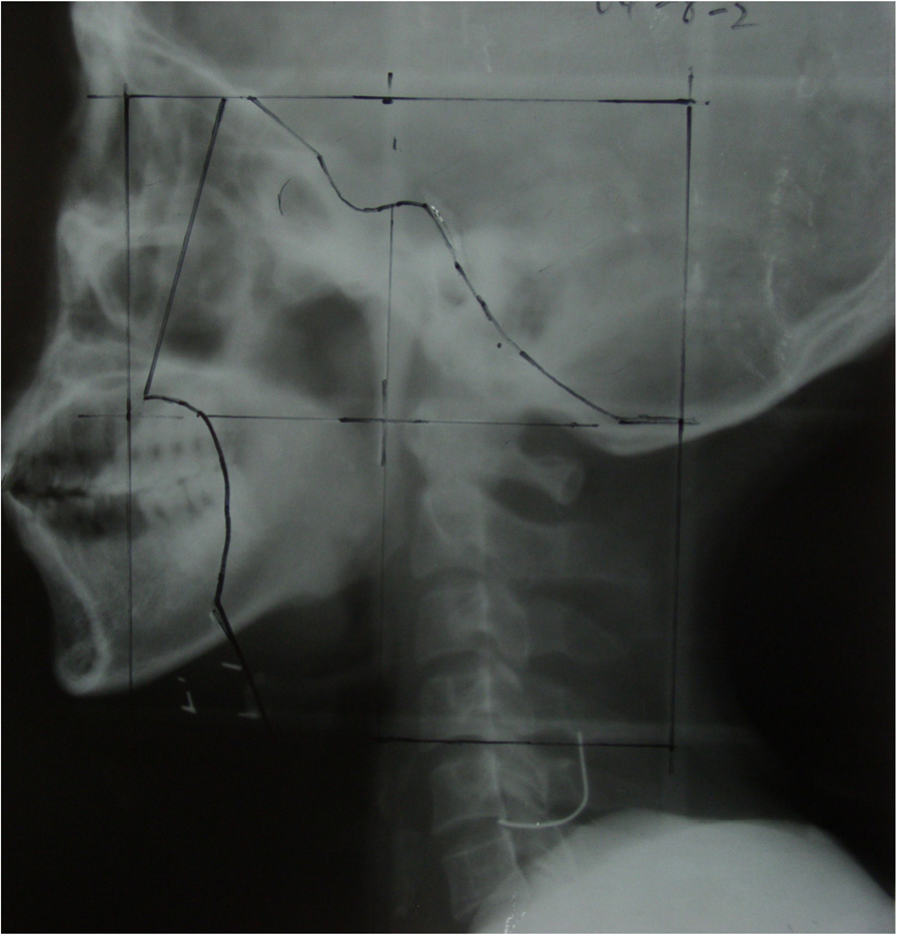
X-ray showing the simulation of the facial and cervical joint field positioning. The transferred submandibular is outside of the irradiation field.

**Figure 6:**
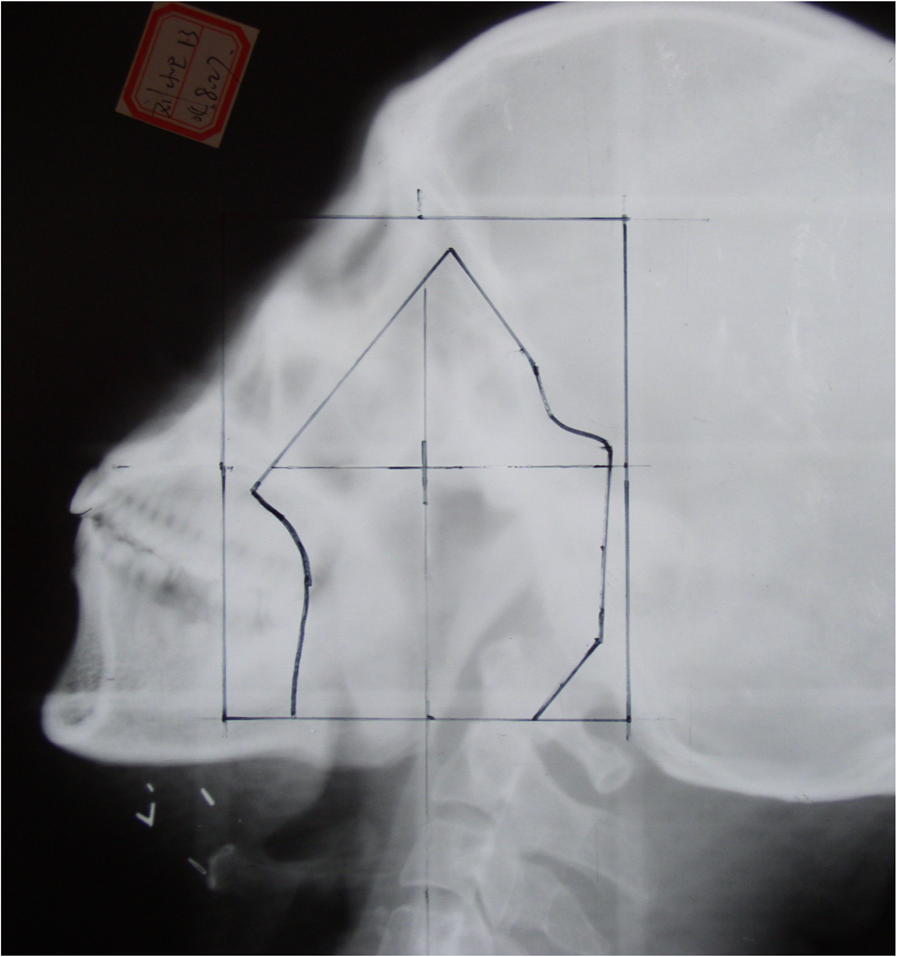
X-ray showing the delineation of the auriculotemporal field in the facial and cervical regions after 19 fractions of radiotherapy (38 Gy); the transferred submandibular gland is outside of the irradiation field.

**Figure 7:**
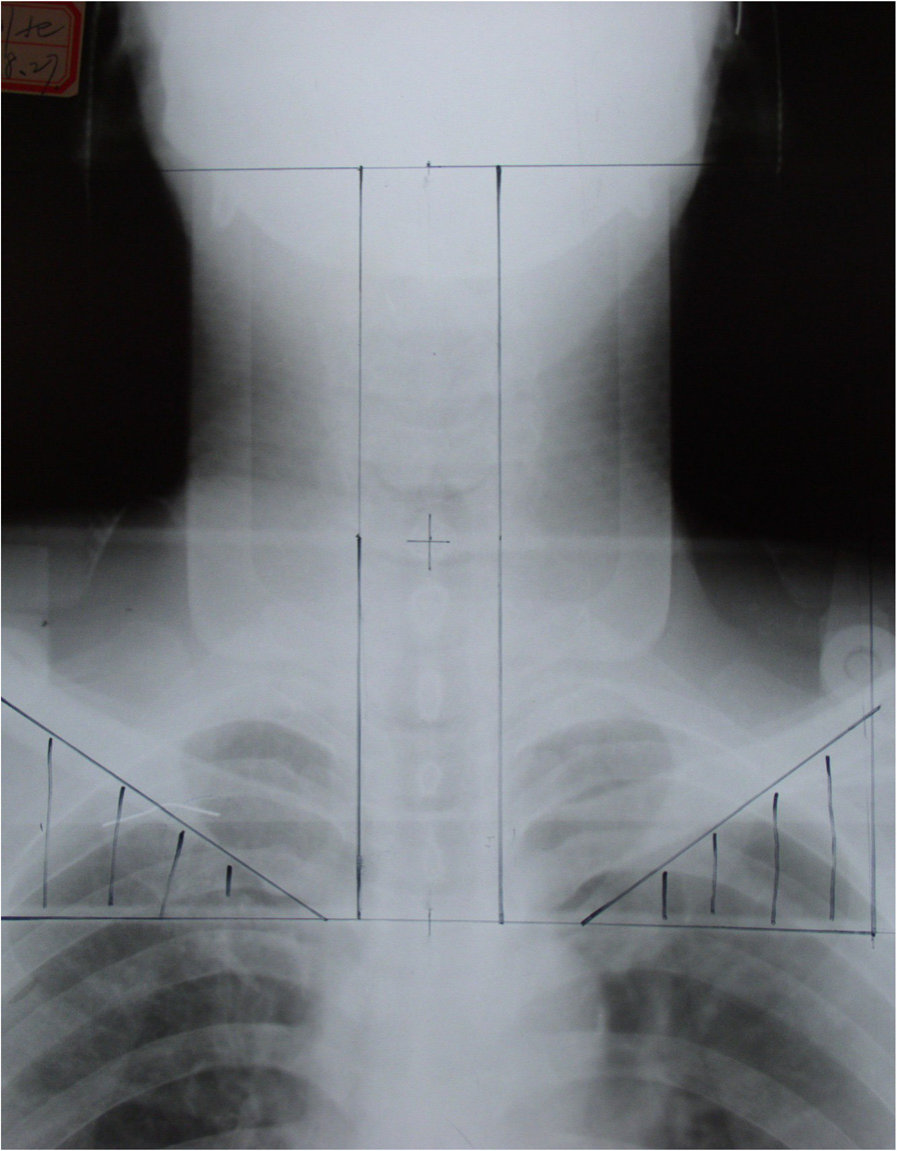
X-ray showing the delineation of the whole neck tangential field in the facial and cervical regions after 19 fractions of radiotherapy (38 Gy); the transferred submandibular gland is shielded.

RT was administered to the experimental and control groups using a Siemens linear accelerator that generated 6 MV X-rays. The position of the center of the irradiation field was unchanged, fixed thermal plastic masks were used, and a low-melting lead shield was employed to specifically block the exposure to radiation by tissues outside of the irradiation field. Conventional fractionated RT was delivered in 2 Gy fractions, once daily, and 5 times per week; the nasopharynx target therapeutic dose was 65–75 Gy, the cervical lymph node metastases therapeutic dose was 60–70 Gy, and the cervical lymph node preventive dose was 45–50 Gy (Figures 5, 6 and 7).

### Observed indicators

#### Postoperative observation

Observation included wound healing after surgery, the occurrence of complications, and submental and submandibular lymph node pathology.

#### Examination of submandibular gland function

Imaging of the submandibular gland was performed at 2 weeks after RT in the experimental group; submandibular gland 99 mTc radionuclide imaging was also performed at 60 months after RT. Imaging of the submandibular gland was performed at 3 days before RT in the control group; submandibular gland 99 mTc radionuclide imaging was also performed at 60 months after RT.

#### Determination of saliva secretion

Saliva secretion was measured in the experimental and control groups at 3, 6, 12, and 60 months after RT. Patients were asked to raise the tip of their tongue against the hard palate, then a number of dry cotton balls were placed in their sublingual mound openings. About 10 minutes later, the wet cotton balls containing saliva were removed and weighed. To reduce physiological interference, determination of saliva secretion was conducted at 1 hour after eating. The amount of saliva secreted was determined as follows: saliva weight (g) = wet cotton ball weight - dry cotton ball weight.

#### Xerostomia questionnaire

In the experimental and control groups, a questionnaire regarding the degree of xerostomia was completed by the patients at 3, 6, 12, and 60 months after RT, and evaluated using the visual analysis scale (VAS) method [7], and by evaluation of subjective symptoms [8]. Xerostomia evaluation criteria refer to the RTOG/EORTC [9] RT staging criteria.

#### Neck lymph node residual rate, recurrence, and survival

At 3 months after RT, the cervical lymph node residual rate, the neck lymph node recurrence site, the cervical lymph node recurrence rate, and the 5-year survival rates were evaluated in the two patient groups.

### Statistical methods

The SPSS 14.0 statistical package was used for processing data. The 5-year survival rate was calculated using the Kaplan-Meier method, and the difference between the groups was compared using the log-rank parallel test. The xerostomia survey data and the submandibular gland radionuclide scintigraphy data for patients at 60 months after RT were compared using the two sample data non-parametric test. Differences in saliva secretion, dry mouth, and the VAS scoring results between the two groups were evaluated using the two independent sample data *t* tests. Survey data, the incidence of severe xerostomia, and the cervical lymph node recurrence rate were compared using the *χ*^*2*^ test. A *P* value of <0.05 was considered statistically significant.

## Results

### Postoperative observation

The submandibular glands of 32 patients were transferred successfully, and no surgery related deaths or complications occurred. All incisions healed well and patient chins were slightly plump after the removal of stitches. A total of 107 lymph nodes were scavenged; metastasis was not found in the intraoperative rapidly frozen or paraffin sections. During the follow-up period, no patients complained of obvious discomfort caused by the surgery.

### Examination of submandibular gland function

Before RT, the submandibular gland secretion function was normal, as confirmed using imaging in the two patient groups; there was no significant difference between the two groups (*P* = 0.675). At 60 months after RT, all of the patients underwent submandibular gland radionuclide scintigraphy; submandibular gland uptake and secretion were significantly higher in the experimental group than in the control group (*P* < 0.001).

### Comparison of saliva flow measurement results

At 3, 6, 12, and 60 months after RT, the average amount of saliva was 1.19 g, 1.28 g, 1.39 g, and 1.60 g, respectively in the experimental group. The average amount of saliva in the control group at 3, 6, 12, and 60 months after RT was 0.58 g, 0.63 g, 0.66 g, and 0.68 g, respectively.

### Evaluation of xerostomia at 3, 6, and 12 months after RT

The incidence of xerostomia in the experimental group at 3 months after RT ranged from moderate to severe, and it was significantly lower than in the control group (37.4% and 93.9%, respectively; *P* < 0.001). The incidence of xerostomia in the experimental group at 6 months after RT ranged from moderate to severe, and it was significantly lower than in the control group (28.1% and 87.9%, respectively; *P* < 0.001). The incidence of xerostomia in the experimental group at 12 months after RT ranged from moderate to severe, and it was significantly lower than in the control group (18.7% and 81.8%, respectively; *P* < 0.001). Thus, at 3, 6, and 12 months after RT, the average xerostomia level in the experimental group was significantly lower than in the control group (Tables 2, 3 and 4). With increasing time after RT, the symptoms of xerostomia progressively improved.

**Table 2:** Survey results regarding the degree of xerostomia at 3 months after RT

**Degree of xerostomia**	**Experimental group**	**Control group**	**Z-value**	**P *- *** **value**
None (G1)	7 (21.9%)	0 (0.0%)	4.428	0.000
Mild (G2)	13 (40.6%)	2 (6.1%)		
Moderate (G3)	8 (25.0%)	18 (54.5%)		
Severe (G4)	4 (12.5%)	13 (39.4%)		

**Table 3:** Survey results regarding the degree of xerostomia at 6 months after RT

**Degree of xerostomia**	**Experimental group**	**Control group**	**Z-value**	**P-value**
None (G1)	8 (25.0%)	0 (0.0%)	4.584	0.000
Mild (G2)	15 (46.9%)	4 (12.1%)		
Moderate (G3)	6 (18.7%)	19 (57.6%)		
Severe (G4)	3 (9.4%)	10 (30.3%)		

**Table 4:** Survey results regarding the degree of xerostomia at 12 months after RT

**Degree of xerostomia**	**Experimental group**	**Control group**	**Z-value**	**P-value**
None (G1)	8 (25.0%)	0 (0.0%)	4.896	0.000
Mild (G2)	18 (56.3%)	6 (18.2%)		
Moderate (G3)	4 (12.5%)	19 (57.%)		
Severe (G4)	2 (6.2%)	8 (24.2%)		

### Evaluation of xerostomia at 60 months after RT

The survey results regarding xerostomia are detailed in Table 5. The incidence of xerostomia in the experimental group ranged from moderate to severe and was significantly lower than in the control group (15.4% and 76.9%, respectively; *P* < 0.001). The estimated VAS score results based on the patients’ own subjective feelings regarding xerostomia were as follows: the VAS score in the experimental group (3.7) was lower than in the control group (5.8), and there were significant differences between the two groups (*P* < 0.001). Some of the important issues concerning the survey results and the degree of subjective feelings regarding the symptoms of xerostomia are presented in Table 6. In the experimental group, speech, chewing, swallowing, changes in eating habits, nighttime xerostomia, the need to wake up to drink frequently, and sleep quality were all improved significantly relative to the control group.

**Table 5:** Survey results regarding the degree of xerostomia at 60 months after RT

**Degree of xerostomia**	**Experimental group**	**Control group**	**Z-value**	**P-value**
None (G1)	7 (26.9%)	0 (0.0%)	4.423	0.000
Mild (G2)	15 (57.7%)	6 (23.1%)		
Moderate (G3)	3 (11.5%)	16 (61.5%)		
Severe (G4)	1 (3.9%)	4 (15.4%)		

**Table 6:** Evaluation of subjective symptoms at 60 months after RT

**Symptoms**	**Experimental group**	**Control group**	**P-value**
Day xerostomia	16 (61.5%)	26 (100%)	0.000
Night xerostomia	4 (15.4%)	20 (76.9%)	0.000
Painful mouth and tongue	6 (23.1%)	9 (34.6%)	0.358
Dry, cracking and painful lips	5 (19.2%)	7 (26.9%)	0.510
Regular drinking during the day	17 (65.4%)	26 (100%)	0.003
Regular drinking during the night	1 (3.9%)	4 (15.4%)	0.347
Drinking when eating	4 (15.4%)	20 (76.9%)	0.000
Difficulty speaking	2 (7.7%)	14 (53.8%)	0.000
Difficulty chewing	3 (11.5%)	17 (65.4%)	0.000
Difficulty swallowing	1 (3.8%)	5 (19.2%)	0.193

### Cervical lymph node residual rate, cervical lymph node recurrence rate, and 5-year survival rate

The experimental and control groups had no cervical lymph node residues at 3 months after RT. The cervical lymph node recurrence rates in the experimental and control groups were 15.6% (5/32) and 12.1% (4/33), respectively; there was no significant difference (*P* = 0.960) between the two groups. Lymph node recurrence occurred exclusively in Zone II and was not evident in Zone I. In the experimental and control groups, the 5-year survival rates were 81.3% (26/32) and 78.8% (26/33), respectively; there was no significant difference between the two groups (*P* = 0.806).

### Follow-up

The follow-up period began at the end of RT and continued until December 31, 2011. A total of 65 patients were followed up for 28–60 months.

## Discussion

The submandibular gland is a mixed gland, containing both serous and mucous cells. Approximately 90% of total saliva is produced by the submandibular gland in non-stimulated conditions. The total daily flow of saliva from the submandibular gland is around 200–300 ml. A considerable proportion of the saliva is mucus; this contributes to the sensation of oral comfort as a result of the continuous lubrication of the oral mucosa. Saliva also lubricates the mouth during periods of non-eating, and reduces the symptoms of xerostomia [10-13].

Three major salivary glands are located in the radiation field when NPC patients receive conventional RT, and radiation damage to these glands is permanent. The basic feature of submandibular gland transfer for the prevention of xerostomia is the transfer of the submandibular gland to the submental area. A stopper is set in the submental area, and only approximately 5% of the total radiation dose (30–32.5 Gy) is received by the submandibular gland; thus, the submandibular gland remains free from damage [14]. Therefore, to preserve submandibular gland function, patients with serious damage and lymph node metastasis in region I were not suitable for surgery. In addition, patients who had undergone previous neck surgery and had a history of RT were not suitable for surgery. In the group of 32 patients that received intraoperative cleaning of the 107 lymph nodes, observation of intraoperative rapidly frozen slices and postoperative paraffin biopsy did not reveal metastasis. Consequently, the prerequisite for submandibular gland transfer in patients with NPC was the absence of neck Zone I lymph node metastasis. In the case of cervical lymph node metastasis, the side of the submandibular gland with no lymph node metastasis was transferred. If there was no lymph node metastasis, the contralateral submandibular gland was transferred.

The key success regarding submandibular gland transfer surgery for the prevention of RT xerostomia lies in patient survival and the sustainability of saliva secretion. In our study, at 60 months after RT, the xerostomia survey results indicated that 26 patients in the control group had varying degrees of xerostomia, with 76.9% of the patients suffering from moderate to severe dry mouth; these results are similar to those reported in the literature [15]. At 3 months after RT, the incidence of xerostomia in the experimental group ranged from moderate to severe and was significantly lower than in the control group; with increasing time after RT, their symptoms improved. In the experimental group at 60 months after RT, the average quantity of saliva secretion, checked using submandibular gland radionuclide scintigraphy, was obviously higher than in the control group; there were significant differences between the two groups. In the experimental group, the incidence of moderate to severe xerostomia, standard of speech, chewing ability, swallowing ability, changes in eating habits, nighttime xerostomia, need to wake up and drink frequently, sleep quality, and VAS scores were significantly improved relative to the control group. As a result of transfer after RT, the submandibular gland not only survived but also retained a strong secretion function. Thus, submandibular gland transfer was an effective method for preventing xerostomia after RT.

In NPC patients at 3 months after RT, the experimental group and the control group had no residual lymph nodes, and submandibular gland transfer did not affect the cervical lymph node control rate. There was no significant difference between the experimental and control groups regarding the cervical lymph node recurrence rate. Recurrence occurred in Region II but not in Region I. Therefore, submandibular gland transfer did not affect the recurrence rate in the cervical lymph node. There was no significant difference in the 5-year survival rate between the two patient groups and submandibular gland transfer did not affect the 5-year survival rate. Thus, submandibular gland transfer had no effect on the long-term efficacy of NPC treatment using RT. These results are consistent with those of Seikaly et al. [16]. These authors reported that when the submandibular gland was transferred to the submental area of the oropharynx, larynx, and hypopharynx during the treatment of malignant tumors using RT, xerostomia did not influence the survival rate or recurrence rate in the lymph nodes of the neck.

Because the transferred submandibular gland could survive after RT, it retained a strong secretory function and could effectively prevent xerostomia after RT. However, submandibular gland transfer could not fully solve the problem of xerostomia because mild and severe xerostomia still occurred. After 5 years, xerostomia still had an occurrence rate of 15.4%. A number of studies [17-19] have shown that when using intensity-modulated RT techniques, the radiation dose delivered to the parotid gland and high-dose irradiation volume can be reduced to protect the functions of the parotid gland. This approach could reduce the degree of xerostomia after RT in NPC patients. Consequently, whether submandibular gland transfer and intensity-modulated RT should be adopted for the protection of the submandibular gland and the prevention of xerostomia after RT in the treatment of NPC requires evaluation in further clinical studies.

## Competing interests

The authors declare that they have no competing interests.

## Authors’ contributions

XMZ and SJL are responsible for the study design. XMZ, FLL and XLL performed the experiments and draft the manuscript. LJY and WW collected the data. HXW and FFX participated in the data analysis and interpretation. All authors read and approved the final manuscript.
